# Theoretical and Experimental Insights into the Chemiresistive Sensing Response of Graphene Quantum Dots: The Role of Oxygen Functional Groups

**DOI:** 10.1021/acsomega.4c08588

**Published:** 2025-02-21

**Authors:** Bruno S. Sampaio, Murilo H. M. Facure, Rafaela S. Andre, Daniel S. Correa, Tiago V. Alves, Luiza A. Mercante

**Affiliations:** † Instituto de Química, Universidade Federal da Bahia (UFBA), Salvador, BA 40170-115, Brazil; ‡ Nanotechnology National Laboratory for Agriculture (LNNA), Embrapa Instrumentação, São Carlos, SP13560-970, Brazil

## Abstract

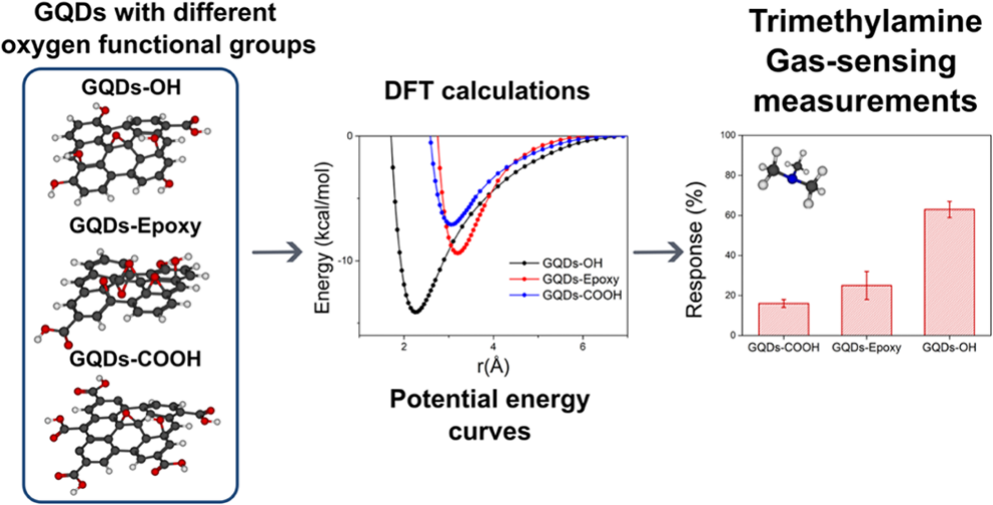

Developing sensitive sensors to trimethylamine (TMA) remains a topic of great interest in areas such as food quality analysis and disease biomarkers. To address this issue, chemiresistive sensors were proposed using graphene quantum dots (GQDs) with different proportions of hydroxyl (GQDs-OH), epoxy (GQDs-epoxy), and carboxyl (GQDs-COOH) groups. These materials exhibited different sensitivities to TMA, with GQDs-OH being the most sensitive, presenting a detection limit of 0.3 ppm and a response of about 4 and 2.5 times higher than those of GQDs-COOH and GQDs-Epoxy, respectively. This difference in sensitivity was elucidated by building, based on density functional theory calculations, potential energy curves of the interaction between TMA and three GQD models. Noncovalent interaction and atoms in molecular analysis were also used to explain the difference in interaction in each model. Our results highlight that the proportion of the oxygen functional groups has a major role in modulating the sensitivity against TMA, with the hydroxyl group providing the greater sensitivity. This was elucidated through computational simulations, which also explained the lower sensitivity of the other materials. Our work serves as a practical guide, demonstrating the importance of coupling computational and experimental methods to achieve a deeper understanding of sensing results.

## 1 Introduction

The detection of certain volatile amines is a task that has attracted much attention in many fields.^1^ Among these compounds, trimethylamine (TMA) is a gas^2,3^ generally used as an indicator for meat quality evaluation and disease diagnosis.^3,4^ For the detection of this harmful volatile, chemiresistive gas sensors have sparked considerable interest^5^ due to their high sensitivity, ease of fabrication, simple operation, and low price.^6^ For this purpose, different materials, including metal oxide semiconductors,^7,8^ conducting polymers^9,10^ and carbon-based materials,^11,12^ have been successfully employed to develop gas sensors.

Graphene quantum dots (GQDs) are a type of carbon-based nanomaterial promising for sensing applications due to their high surface-to-volume ratio, low cost, and remarkable surface functionalities.^13^ Moreover, different functional groups on the GQD structures provide effective adsorption sites for interacting with the gaseous analyte, improving the sensor response.^14^ For this reason, different experimental approaches have been developed to modify the structural properties of GQDs and, consequently, improve their reactivity and sensitivity toward toxic gases.

However, improving the performance of chemical sensors through experimentation alone can be highly time-consuming and resource-intensive. This challenge can be mitigated by integrating experimental approaches with density functional theory (DFT) calculations.^15^ Besides saving time, DFT calculations can be effectively used as a predictive tool for rationalizing novel gas sensors with improved sensitivity. Furthermore, DFT-based simulations provide critical information for understanding the interaction between the sensing layer and the analyte under investigation at the molecular level, which can help understand the experimental results.^16^

Arunragsa et al.,^17^ for instance, reported the use of self-consistent charge density functional tight binding (SCC-DFTB) calculations to describe the interaction mechanism between ammonia (NH_3_) molecules and GQDs. They concluded that the interaction between ammonia and the edge hydroxyl groups presented the highest interaction energy, which shows that the edge functionalization with hydroxyl groups is a good strategy to improve the sensor’s performance toward NH_3_ detection.

In this context, the number of steps required to improve the sensitivity of a gas sensor can be reduced by using computational tools. In our previous work,^18^ three GQD structures with different proportions of hydroxyl, epoxy, and carboxyl groups were obtained using a hydrothermal method and employed for the optical detection of Fe^3+^ ions. In this work, we aim to assess the impact of oxygen functional groups on the sensitivity of GQDs for TMA detection using DFT calculations. To the best of our knowledge, this is the first study to systematically evaluate the effect of each functional group of a GQD to optimize the sensitivity of a GQD-based electrical sensor for TMA detection.

## 2 Experimental Section

### 2.1 Synthesis of the GQDs

GQDs were synthesized following a hydrothermal procedure fully described in our previous work,^18^ but a few details will be given here. The synthesis was performed in a Teflon-lined stainless-steel autoclave (50 mL) by heating 40 mL of the graphene oxide (GO) dispersion at 1 °C min^–1^ for 10 h. Three distinct syntheses were carried out by varying the temperature (130, 160, and 190 °C), GO concentration (1.25, 2.00, and 2.75 mg L^–1^), and pH (4.5, 8.0, and 9.5). The resulting products were filtered by using a 0.22 μm micropore syringe filter, and the obtained dispersions were then freeze-dried to obtain the GQD powder. The full characterization of the obtained materials, reported in our previous paper,^18^ showed that these reaction conditions led to GQDs with similar sizes and thicknesses and distinct proportions of different oxygen functionalities: one with a greater proportion of hydroxyl groups, another with a higher proportion of epoxy groups, and a third one with a greater proportion of carboxyl groups. Hereafter, these GQD dispersions will be termed GQDs-1, GQDs-2, and GQDs-3, respectively.

### 2.2 Computational Studies

DFT calculations were employed to obtain potential energy curves (PECs) and quantify the interactions through rigid scan calculations to evaluate the interactions of the TMA with the different GQD structures. To represent the three GQDs of our previous work,^18^ three models were built: one with only hydroxyl groups (GQDs-OH), one with only epoxy groups (GQDs-Epoxy), and one with only carboxyl groups (GQDs-COOH). The acronyms of the computational and experimental models are shown in Table 1.

**Table 1:** Acronyms of Computational Models and Experimental GQDs

main functional group	computational model	experimental GQDs
OH	GQDs-OH	GQDs-1
Epoxy	GQDs-Epoxy	GQDs-2
COOH	GQDs-COOH	GQDs-3

The geometry optimizations and rigid scans were performed using the M06-2X^19^ functional and the 6-31+G(d,p) basis set, as implemented in the Gaussian 09^20^ suite of programs. The confirmation of the nature of the stationary points was based on an analysis of the harmonic vibrational frequencies calculated at the same level of theory. The adsorption sites for constructing the PECs were chosen based on the molecular electrostatic potential maps (MEPs), as obtained with the Jmol software.^21^ This analysis allowed us to evaluate the molecular sites of the highest electron-withdrawing character. Three sites for each structure were chosen to simulate the adsorption, thus meaning that nine complexes were obtained.

The PECs were constructed through the interaction between the GQDs and the nitrogen of the TMA molecule in the ranges 1.70 and 7.10 Å between the fragments. After determining the equilibrium internuclear distance (*r*_e_), 10 additional points were calculated around this region with a step size of 0.05 Å. After investigating the possible ways that the TMA molecule can approach the GQDs, we found the most stable structures by approaching the TMA molecule above the GQD surface, with the partially negatively charged nitrogen facing the adsorption sites. Based on this, this approach was fixed for all scans. To explain the difference in the stability of each complex, atoms in molecules (AIM) topological parameters and noncovalent interaction (NCI) analysis were performed using the Multiwfn 3.8 software.^22^

### 2.3 TMA Sensing Measurements

The sensors were prepared by drop-casting 5 μL of a GQD suspension (5 mg mL^–1^) onto a gold-interdigitated electrode (IDE) surface and left to dry at room temperature. Gas sensing measurements were carried out at room temperature (25 ± 2 °C) in a homemade chamber.^23^ The gas sensing performance was evaluated by exposing the sensors to various concentrations of TMA (1–50 ppm) and analyzing the changes in electrical resistance using an impedance analyzer (Solartron, model 1260). The data were collected in the frequency range from 1 Hz up to 1 MHz, using an AC-applied voltage of 75 mV. The test chamber’s relative humidity (RH) was kept at around 50% by using the saturated Mg(NO_3_)_2_ solution.^24^ The sensor response was defined as response (%) = [((*R*_a_ – *R*_g_)/*R*_a_)] × 100, where *R*_a_ is the electrical resistance in air and *R*_g_ is the electrical resistance after exposure to the gas. The sensors obtained with the GQD-1, 2, and 3 products will also be referred to by their main functional group (Table 1), i.e., GQDs-OH, GQDs-Epoxy, and GQDs-COOH, respectively.

## 3 Results and Discussion

### 3.1 Computational Simulations

**Figure 1:**
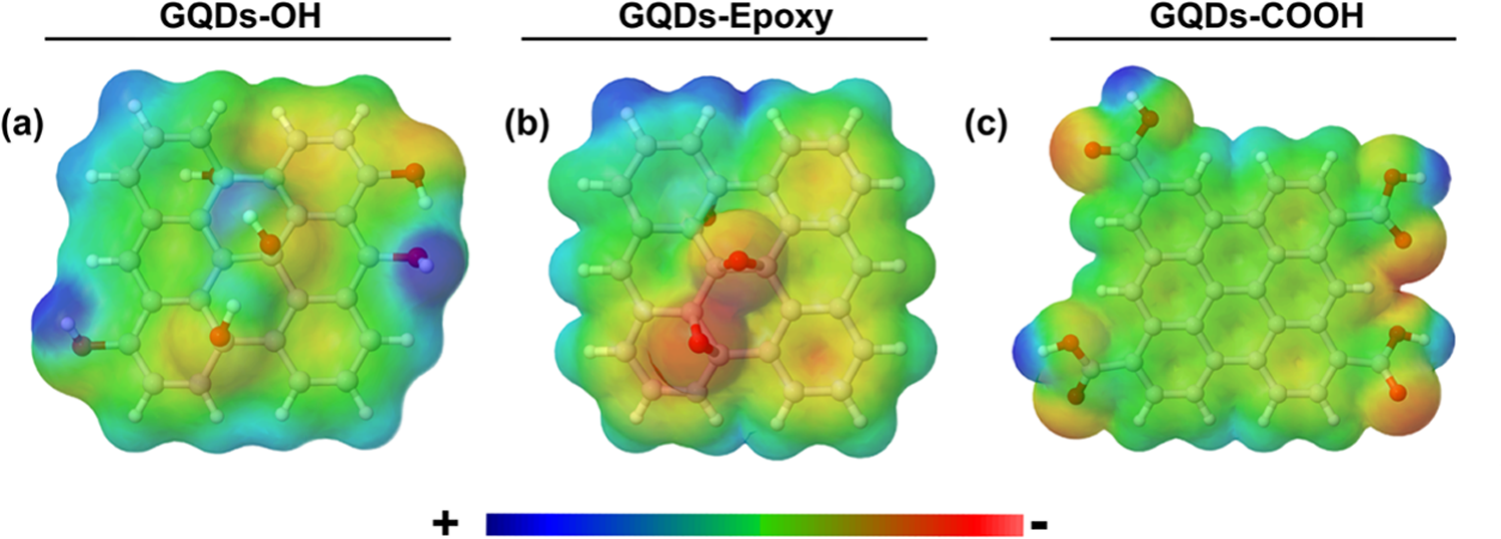
Electrostatic potentials plotted in electron density isosurfaces for the structures: (a) GQDs-OH, (b) GQDs-Epoxy, and (c) GQDs-COOH.

The Cartesian coordinates of TMA and functionalized GQDs and their lowest vibrational frequencies are listed in Table S1. All stationary points are characterized as minima; i.e., they were identified as having no imaginary frequencies. To select the adsorption site in which the TMA will interact, MEPs were obtained to highlight the regions of the lowest electron density, which could act as electron acceptors, as illustrated in Figure 1.

**Figure 2:**
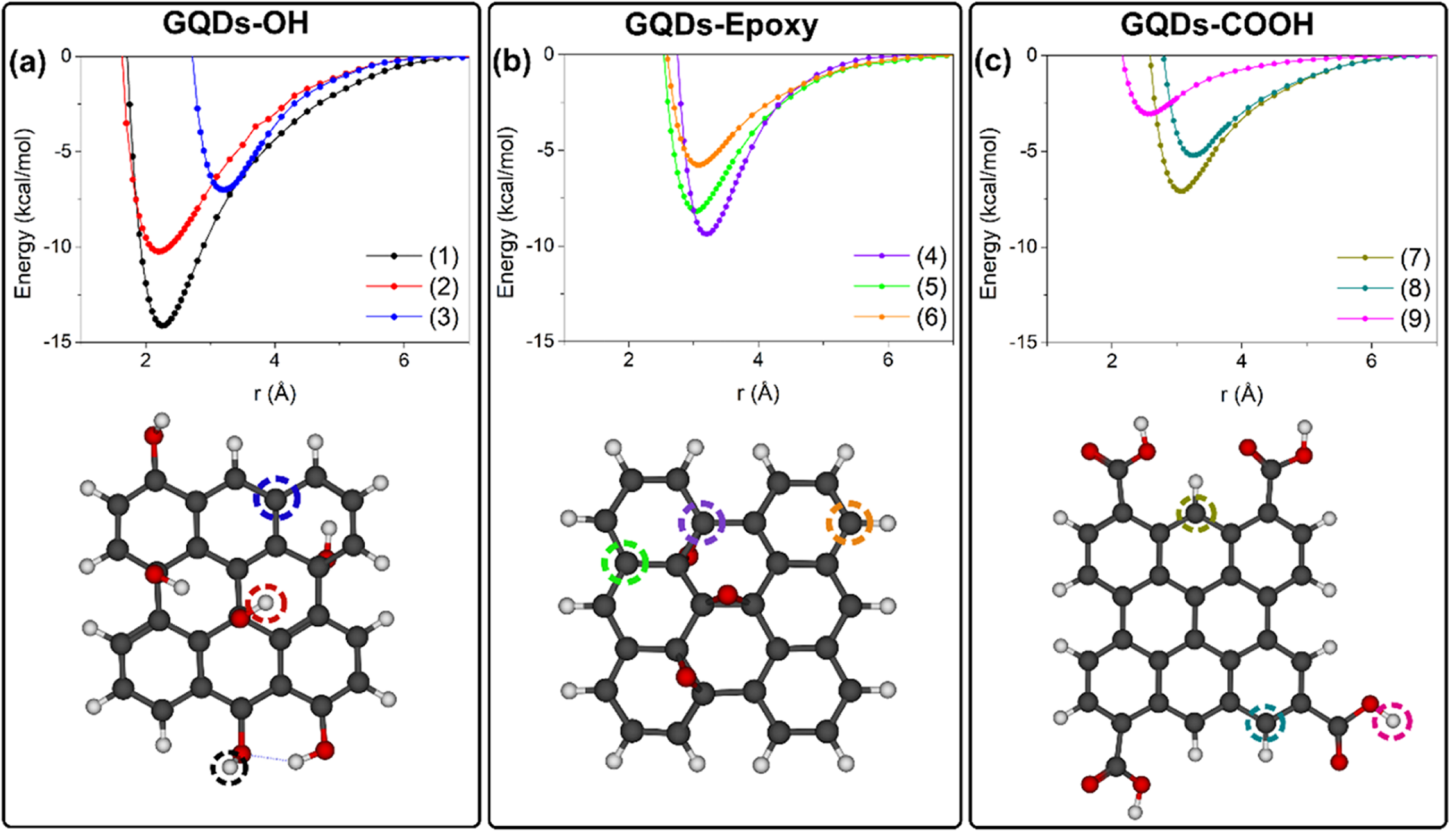
Potential energy curves and selected adsorption sites in the model of (a) GQDs-OH at the edge hydroxyl (1), basal hydroxyl (2), and carbon (3); (b) GQDs-Epoxy at carbons with different proximities to the epoxy groups (4)–(6); (c) GQDs-COOH at a carbon neighbor to two COOH groups (7) and to one COOH (8) and at an O–H of the COOH group (9).

For the GQDs-OH, the MEPs suggest three regions with low electron density at (1) the hydroxyl group at the edge, (2) the basal hydroxyl group, and (3) the carbon close to the basal hydroxyl group. In the case of the GQDs-Epoxy, the low electron density regions were identified over the carbons close to the epoxy groups, shown in adsorptions (4) and (5), respectively. A carbon center on the opposite side of the epoxy groups (6) was also considered due to the moderate electron density observed in the MEP and to evaluate the effect of the proximity to the epoxy group on the interaction between the fragments. For the GQDs-COOH, the low electron density regions were mainly observed in the hydroxyl, followed by two carbon centers at the edges of the material, adsorbed in (7), (8), and (9), respectively. Considering these adsorption sites, we constructed the potential energy curves of the interaction between TMA and each site constructed. The obtained PECs, the GQD models, and the selected adsorption sites are shown in Figure 2.

Table S2 lists all of the electronic energies as a function of the internuclear distances of the (1)–(9) scans, calculated at the M06-2X/6-31+G(d,p) level of theory. The equilibrium internuclear distances (*r*_e_) between the GQDs and TMA and the dissociation energies (*D*_e_) estimated in the asymptotic limit are listed in Table 2. The top and side views of the scans reported in Table 2 are represented in Figure S1.

**Table 2:** *r*_e_ (in Å) and *D*_e_ (in kcal/mol) Values, Calculated at the M06-2X/6-31+G(d,p) Level of Theory

complex	*r*_e_ (Å)	*D*_e_ (kcal/mol)
GQDs-OH	2.25	14.13
GQDs-OH	2.20	10.23
GQDs-OH	3.20	7.00
GQDs-Epoxy	3.20	9.38
GQDs-Epoxy	3.05	8.19
GQDs-Epoxy	3.10	5.78
GQDs-COOH	3.05	7.09
GQDs-COOH	3.30	5.19
GQDs-COOH	2.55	3.04

The *r*_e_ values obtained for the interaction of the TMA molecule with hydroxyl groups (1, 2, and 9) were between 2.20–2.55 and 3.05–3.30 Å when the interactions occur through carbon atoms. Similar values are reported in the literature. For instance, Arunragsa et al.^17^ obtained a value of 2.00 and 3.20 Å for the interaction of NH_3_ with the edge hydroxyl and basal carbon of GQDs, respectively. At the ωB97XD/6-31G(d,p) level of theory, de Menezes et al.^25^ determined a value of 3.15 Å for the interaction of ammonia with the basal carbon of a pristine GQD. In the following sections, we will discuss the results obtained in the PECs for each structure and perform a topological analysis to explain the difference in the *D*_e_ values for each site.

#### 3.1.1 Interaction Between GQDs-OH and TMA: PECs and Topological Analysis

**Figure 3:**
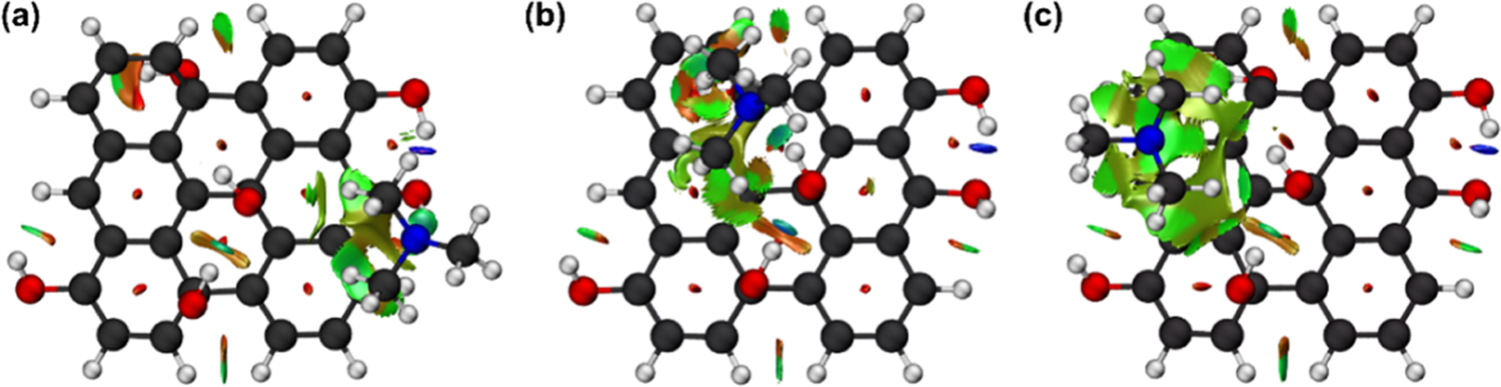
NCI analysis of the interactions between the TMA and the GQDs-OH at (a) the edge hydroxyl (1), (b) basal hydroxyl (2), and (c) carbon (3). Isosurface contour value = 0.50 au.

In the GQD-OH case, strong adsorbate molecule interactions with the hydroxyl group at the edge (1) and basal (2) regions were observed. The equilibrium distances of 2.25 and 2.20 Å suggest a quasi-covalent character between the TMA and GQDs-OH, with *D*_e_ values of 14.13 and 10.23 kcal/mol, respectively. Comparatively to the region (1), the adsorption on (3) depicts a shallower well and an equilibrium distance 0.95 Å larger. To explain the difference in the energy between these adsorptions, the nature of the interactions in the GQDs-OH structure was evaluated by using NCI analysis (Figure 3).

**Figure 4:**
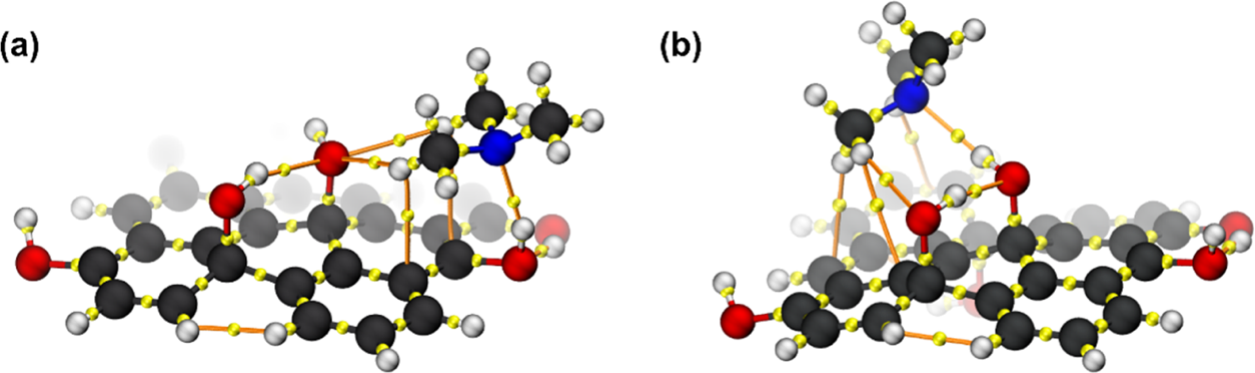
[3, −1] Critical points and bond paths for the interaction between TMA and GQDs-OH at the (a) edge hydroxyl and (b) basal hydroxyl.

The blue and green colors in the isosurfaces represent strong attractive and van der Waals (vdW) interactions, respectively, while red represents repulsive interactions. As shown in Figure 3a, a H-bonding interaction is established between the nitrogen of the TMA and the edge hydroxyl of the GQDs-OH. This interaction is also stabilized by the vdW interaction with the carbons of the GQD sheet. These same interactions are also observed with the hydroxyl group of the basal region (Figure 3b). A topological analysis was performed to compare these hydrogen bond interactions and quantify the values of electron density (ρ) and the Laplacian of the electron density (∇^2^ρ) at the [3, −1] critical point (C.P) between TMA and the edge and basal hydroxyl (Figure 4). These quantities present a linear relationship with the hydrogen bond strength and can be used to compare the difference in the interaction for each site in the GQDs-OH structure.^26^ To properly quantify these values, the structures of the rigid scan at the *r*_e_ of (1) and (2) were optimized at the same level of theory. The Cartesian coordinates of the equilibrium structures are shown in Table S3.

The ρ values obtained for the edge and basal hydroxyls were 0.031 and 0.027 au, and the ∇^2^ρ values were 0.077 and 0.072 au, respectively. For both, our results are within the range expected for hydrogen bond interactions, i.e., 0.002–0.034 au for ρ and 0.024–0.139 au for the ∇^2^ρ.^27^ As expected, the interaction of H-bonding with the edge hydroxyl is stronger when compared with that of the basal hydroxyl, stabilizing (1). For (3), the TMA adsorption with the GQD sheet is only stabilized by vdW interactions, explaining the shallower well discussed before. Considering the strength of the interaction with TMA in a structure with a greater degree of hydroxyl groups, the H-bond interactions are the main pathway for stabilizing this complex, especially at the edge.

#### 3.1.2 Interaction Between GQDs-Epoxy and TMA: PECs and Topological Analysis

**Figure 5:**
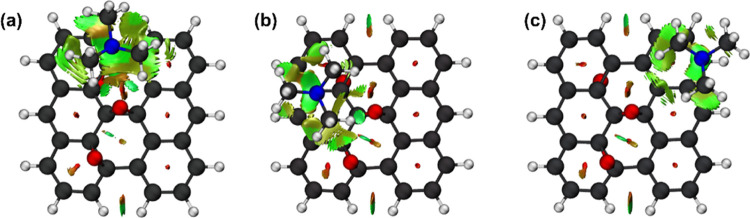
NCI analysis of the interactions between the TMA and the GQDs-Epoxy at (a) carbon directly bound to an epoxy group (4), (b) carbon neighboring to an epoxy group (5), and (c) carbon farther from the epoxy groups (6). Isosurface contour value = 0.50 au.

In the GQDs-Epoxy structure, the interaction (4) with the carbon bound to an epoxy group was found to be stronger than with the site (5), which is neighboring an epoxy group (Table 2). When the interaction occurs in the carbon farther away from the epoxy groups, such as in (6), the energy is even lower, indicating that the proximity to the epoxy group plays an important role in stabilizing this structure. Based on the NCI analysis, it can be concluded that the interactions in all GQDs-epoxy structures are mainly driven by vdW interactions between the TMA and the carbon sheet, with some stabilization contributions from the CH···O interactions, as shown in Figure 5.

**Figure 6:**
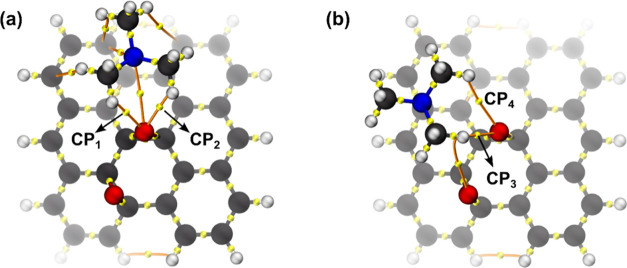
[3, −1] Critical points for the interaction between TMA and GQDs-Epoxy at the (a) epoxy group in (4) and (b) epoxy group in (5).

Based on the MEP (Figure 1), we observed that carbon (4) is slightly more positive than carbon (5), favoring a more significant electrostatic interaction. This effect can be associated with a more electronegative epoxy group, attracting the electron density from the carbon atoms. In carbons (4) and (5), the TMA is also stabilized by two weak C–H···O hydrogen bond interactions. However, in the adsorption site (5), one of the C–H···O interactions yields a smaller isosurface, indicating a smaller electron density and, consequently, a weaker interaction (Figure 5b). To confirm this hypothesis, we estimated the values of ρ at the [3, −1] CP_x_ (*x* = 1, 2, 3, and 4) formed in the CH···O interaction in (4) and (5). These results are illustrated in Figure 6a,b, respectively.

For the interaction with the adsorption site (4) (Figure 6a), CP_1_ and CP_2_ have values for ρ of 0.011 and 0.014 au, respectively. In the case of site (5) (Figure 6b), CP_3_ and CP_4_ present values of 0.013 and 0.004 au, respectively. In fact, for the complex in (5) (Figure 6b), the C–H···O interaction shows a smaller electron density in its internuclear region and, therefore, has a weaker interaction than the others. In this sense, the orientation of the TMA toward the epoxy group should contribute to stabilizing the complex. This was also highlighted by Prasert and Sutthibutpong,^28^ which evaluated the effect of the proximity of ascorbic acid, dopamine, and uric acid molecules to the epoxy group on graphene oxide. The authors highlighted that the regions closer to the epoxy groups were the primary interaction sites. For site (6), the carbon is less positive (Figure 1), and there are no interactions with the epoxy group, which explains the lower energy from the GQDs-Epoxy structures. In this sense, in a structure with a greater proportion of epoxy groups, the interaction with a carbon closer to the epoxy groups is more favored energetically due to the presence of more positive carbon sites and the stabilization provided by the interaction with the epoxy group itself.

#### 3.1.3 Interaction Between GQDs-COOH and TMA: PECs and Topological Analysis

**Figure 7:**
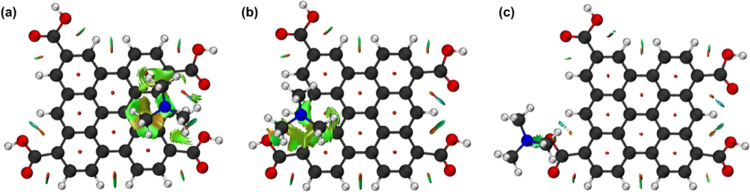
NCI analysis of the interactions between the TMA and the GQDs-COOH at (a) carbon (7) closer to both carboxyl groups, (b) carbon (8) closer to one of the carboxyl groups, and (c) the O–H of one of the carboxyl groups. Isosurface contour value = 0.50 au.

For GQDs-COOH, like in the GQDs-Epoxy case, we also observed that the TMA interacts more energetically with the carbons closer to the functional groups. The internuclear equilibrium distances for (7), (8), and (9) were 3.05, 3.30, and 2.55 Å, respectively. Based on the NCI isosurfaces, it can be concluded that the interactions with this structure are also mainly dominated by the vdW interactions, followed by H-bonding interaction with the hydroxyl of the COOH group, as depicted in Figure 7.

The NCI analysis for these structures shows that in carbon (7), there is a vdW stabilization factor arising from both C-C bonds of the carboxyl groups, and in carbon (8), this same interaction arises from only one group. This factor contributes to the stabilization in (7). Even though in structure (9) there is a H-bond between the TMA and the hydroxyl (Figure 7c), which led to the lowest *r*_e_ among the GQDs-COOH scans, this was the weakest interaction among the scans. The latter can be explained by the directionality of the interaction, which affects the energy value of H-bonds.^29^ Therefore, the interaction of TMA with a carbon closer to the COOH groups is more favored energetically, and the H-bond formation with the hydroxyl of the carboxyl group above the plane does not lead to considerable stabilization.

Based on these results, the GQD structure with a greater proportion of hydroxyl groups interacts more significantly with TMA, followed by that with epoxy groups. In this sense, our computational simulation indicates that the hydroxyl group plays a critical role in the interaction mechanism of GQD and TMA. The role of this group for sensing activity has already been emphasized for other analytes, such as NO_2_^30^ and water vapor.^31^ Besides, the smaller values of *D*_e_ obtained for the GQDs-COOH structure indicate that for GQDs with a greater functionalization of carboxyl groups, the COOH will have a smaller contribution to the stabilization of the GQDs···TMA complex, resulting in a smaller sensitivity.

### 3.2 Experimental Results

**Figure 8:**
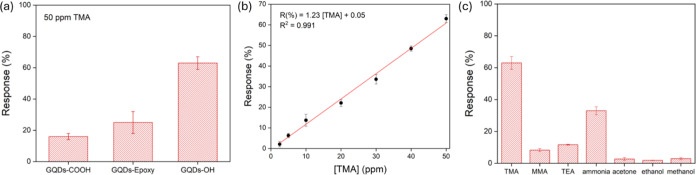
(a) Responses of GQDs-COOH, GQDs-Epoxy, and GQDs-OH sensors to 50 ppm of TMA. (b) Linear relationship between GQDs-OH sensor responses and different TMA concentrations at room temperature. (c) Responses of GQDs-OH sensor versus 50 ppm of various gases (methylamine—MMA, triethylamine—TEA, ammonia, acetone, ethanol, and methanol).

To validate the DFT results, the sensing performance of the GQDs was evaluated as the relative response of the material toward 50 ppm of TMA at room temperature. As shown in Figure 8a, the sensor based on GQDs-OH showed a remarkable response (63%) toward TMA when compared to the GQDs-COOH (16%) and GQDs-Epoxy (25%), suggesting that the sensor presents discrimination ability to detect TMA, corroborating the DFT study.

As shown in Figure 8b, the response of the GQDs-OH sensor toward TMA increases significantly with gas concentration increase, and there is a good linear relationship (*R*^2^ = 0.991) between the response and the concentration of TMA from 1 to 50 ppm. The limit of detection (LOD = 3σ/*S*, where σ is the standard deviation of the response at the lowest concentration, and *S* is the slope of the calibration curve) of GQDs-OH was approximately 0.3 ppm, which is enough for the practical application of seafood freshness detection.^32^

Figure S2a shows the dynamic response curve of the GQDs-OH-based sensor exposed to 50 ppm of TMA at room temperature. During four consecutive testing cycles, the electrical response remained almost unchanged, indicating good repeatability of the sensor. Additionally, Figure S2b shows the response of the GQDs-OH-based sensor for 1 month, which varies in the range of 10%, reflecting the stable detection ability of the proposed sensor.

The comparison with the TMA sensing properties of recently reported gas sensors (shown in Table 3) indicates that the values obtained in this work are comparable to or even superior to those recently reported in the literature. The advantages obtained by using the GQDs-OH for the electrical detection of TMA go beyond the LODs reported here since it is a convenient way to detect amine gas in food samples at room temperature.

**Table 3:** Performance Comparison of GQDs-OH With Other Materials for the Electrical Detection of TMA^a^

material	working temperature	working range (ppm)	LOD (ppm)	references
g-C_3_N_4_/Bi2MoO_6_	RT	5–20	1.3	Wu et al.^33^
graphene-NiGa_2_O_4_	RT		0.01	Chu et al.^34^
MoO_3_/MoSe_2_	RT	0.02–10	0.02	Zhou et al.^35^
NiO/In_2_O_3_	200 °C	0.5–200	0.5	Meng et al.^36^
NiMoO_4_/MoO_3_	200 °C	0.1–100	0.3	Meng et al.^37^
Co_3_O_4_/In_2_O_3_	200 °C	1–100	1.0	Ji et al.^38^
GQDs-OH	RT	1–50 ppm	0.3	this work

a RT, room temperature.

To evaluate the selectivity of the GQD-based sensor, a set of six gases, including methylamine (MMA), triethylamine (TEA), ammonia, acetone, ethanol, and methanol, were tested as interfering substances, as shown in Figure 8c. The response to TMA was about 2–22 times that of other interfering gases, which benefited from the unique molecular recognition caused by the hydrogen-bonding interaction between the hydroxyl oxygen moiety of GQDs and the amino hydrogen group of the target molecule.

According to the computational study, the enhanced sensing response observed for the experimental GQD-OH sensor is mainly related to the interaction of TMA with the edge hydroxyl. Based on the characterization shown in our previous work,^18^ the hydrothermally obtained GQDs-Epoxy and GQDs-COOH also have hydroxyl groups in their structure in a smaller proportion than the GQDs-OH. As shown in Table 2 of the computational study, the interaction with the edge and basal hydroxyl led to the strongest interactions related to the sensing response of GQDs-OH. However, compared with GQDs-Epoxy and GQDs-COOH, the electrical response of the GQDs-OH sensor is 38% and 47% larger, respectively. This difference in the electrical response suggests that the nature of the interaction that led to the electrical response of GQDs-OH is different from that obtained for GQDs-Epoxy and GQD-COOH-based sensors. Therefore, despite the presence of OH in GQDs-Epoxy and GQD-COOH-based sensors, a strong H-bonding interaction with TMA does not explain the sensing results obtained for them. Based on the computational section, the interaction with a carbon of the GQD, closer to the epoxy and carboxyl groups, can better explain the sensing results obtained in Figure 7a for GQDs-Epoxy and GQDs-COOH. Furthermore, based on the theoretical and experimental results, it can be concluded that GQD-based sensors are more sensitive to TMA when there is a higher degree of functionalization with hydroxyl groups. Therefore, to enhance the sensitivity of GQD-based electrical sensors toward TMA, the synthesis parameters should be selected in a way that results in a hydroxyl-rich chemical composition. Other works report the use of different synthetic procedures that can also lead to GQDs with hydroxyl-rich structures. For instance, Yang et al.^39^ and Kappen et al.^40^ reported the use of a photo-Fenton reaction and the pyrolysis method, respectively, to obtain the GQDs with a greater proportion of hydroxyl groups.

## 4 Conclusions

The influence of oxygen functional groups (hydroxyl, epoxy, and carboxyl groups) on the sensitivity of graphene quantum dots (GQDs) toward TMA detection was successfully investigated by theoretical and experimental approaches. Specifically, our results suggested that different proportions of oxygen functional groups led to different sensitivities toward the TMA molecule, with the GQD-OH sensor being the most sensitive, presenting an LOD of 0.3 ppm and an electrical response of 63%. DFT calculations revealed that the greater sensitivity of GQDs-OH toward TMA is mainly due to the interaction with the edge hydroxyl groups of GQDs. Moreover, the results demonstrated that the GQDs-Epoxy sensor is more sensitive to TMA than the GQDs-COOH since the interaction with a carbon closer to an epoxy group led to a greater *D*_e_ value than any interaction in the GQD-COOH structure. In this direction, our findings show that controlling the functionalization of the GQD structure can tune the sensor sensitivity toward a specific analyte. This leaves room for improving GQD-based sensors by focusing on the interplay between the proportions of oxygen functional groups and their corresponding sensing performances. A similar approach to the one used in this work in a doped GQD structure is also an interesting possibility to be considered in future works. Lastly, we emphasize that using computational simulations is a promising strategy to not only understand the sensing results obtained in the experiments but also to predict, by modeling the GQDs, which structure modifications could lead to better sensing performances toward varied analytes.

## Supporting Information Available

Top and side views of the DFT scan in all GQDs, Cartesian coordinates of the theoretical models, electronic energies concerning the distance of the scans, and Cartesian coordinates of the two optimized GQDs-OH···TMA structures, and dynamic response and long-term stability curves for the GQD-OH sensor (PDF)

## References

[ref1] Zhang P.; Wu T.; Cao H.; Zhang J.; James T. D.; Sun X. Fluorometric Detection of Volatile Amines Using an Indanonalkene Platform. Org. Chem. Front. 2023, 10 (6), 1393–1398. 10.1039/D2QO02023H.

[ref2] Zhang W.; Sun Q.; Zhu Y.; Sun J.; Wu Z.; Tian N. High-Performance Trimethylamine Sensor Based on an Imine Covalent Organic Framework. ACS Sens. 2024, 9 (6), 3262–3271. 10.1021/acssensors.4c00613. 38809959

[ref3] Zhang D.; Zhou D.; Mi H.; Wang Z.; Zhang P.; Xi G. Highly Sensitive Trimethylamine QCM Sensor Based on Porous Functionalized Tungsten Disulfide/Polyacrylic Acid Composite for Seafood Freshness Detection. Sens. Actuators, B 2024, 417, 136188 10.1016/j.snb.2024.136188.

[ref4] Ma S.; Guo J.; Zhang H.; Shao X.; Zhang D. A Room Temperature Trimethylamine Gas Sensor Based on Electrospinned Molybdenum Oxide Nanofibers/Ti3C2Tx MXene Heterojunction. Nanomaterials 2024, 14 (6), 537 10.3390/nano14060537. 38535685 PMC10975576

[ref5] Li S.; Zhang L. Accurate First-Principles Simulation for the Response of 2D Chemiresistive Gas Sensors. NPJ Comput. Mater. 2024, 10 (1), 138 10.1038/s41524-024-01329-z.

[ref6] Wei H.; Zhang H.; Song B.; Yuan K.; Xiao H.; Cao Y.; Cao Q. Metal–Organic Framework (MOF) Derivatives as Promising Chemiresistive Gas Sensing Materials: A Review. Int. J. Environ. Res. Public Health 2023, 20 (5), 4388 10.3390/ijerph20054388. 36901399 PMC10001476

[ref7] Li Z.; Zhang D.; Wang X.; Liu X.; Yang Y.; Du C.; Guo J.; Zhang Y. Passive and Wireless NFC Tag-Type Trimethylamine Gas Detection Based on WO3/MXene Composite Sensors. J. Alloys Compd. 2023, 939, 168730 10.1016/j.jallcom.2023.168730.

[ref8] Zhang F.; Liu K.; Li H.; Cui S.; Zhang D.; Zeng J.; Yan Z. MoO3Nanorods Decorated by PbMoO4Nanoparticles for Enhanced Trimethylamine Sensing Performances at Low Working Temperature. ACS Appl. Mater. Interfaces 2022, 14 (21), 24610–24619. 10.1021/acsami.2c04722. 35604024

[ref9] Rath R. J.; Farajikhah S.; Oveissi F.; Shahrbabaki Z.; Yun J.; Naficy S.; Dehghani F. A Polymer-Based Chemiresistive Gas Sensor for Selective Detection of Ammonia Gas. Adv. Sens. Res. 2024, 3 (1), 2300125 10.1002/adsr.202300125.

[ref10] Wen J.; Wang S.; Feng J.; Ma J.; Zhang H.; Wu P.; Li G.; Wu Z.; Meng F.; Li L.; Tian Y. Recent Progress in Polyaniline-Based Chemiresistive Flexible Gas Sensors: Design, Nanostructures, and Composite Materials. J. Mater. Chem. A 2024, 12 (11), 6190–6210. 10.1039/D3TA07687C.

[ref11] Gupta S.; Ravikant C.; Kaur A. Novel Flexible Chemiresistive Ammonia Sensors Based on RGO/ZIF-8 Composites Deposited on Conductive Graphite Sheets. Diamond Relat. Mater. 2024, 148, 111473 10.1016/j.diamond.2024.111473.

[ref12] Kyokunzire P.; Zaraket J.; Fierro V.; Celzard A. Recent Developments in the Use of Activated Carbon-Based Materials for Gas Sensing Applications. J. Environ. Chem. Eng. 2024, 12 (5), 113702 10.1016/j.jece.2024.113702.

[ref13] Thai V.-P.; Tran D. N.; Kosugi K.; Takahashi K.; Sasaki T.; Kikuchi T. One-Step Synthesis of N-Doped Graphene Quantum Dots via Plasma Contacting Liquid for Multiple Heavy Metal Ion Detection. ACS Appl. Nano Mater. 2024, 7 (11), 12664–12672. 10.1021/acsanm.4c01134.

[ref14] Zhu X.; Li Y.; Cao P.; Li P.; Xing X.; Yu Y.; Guo R.; Yang H. Recent Advances of Graphene Quantum Dots in Chemiresistive Gas Sensors. Nanomaterials 2023, 13 (21), 2880 10.3390/nano13212880. 37947725 PMC10647816

[ref15] Yan Z.; Zhang Y.; Kang W.; Deng N.; Pan Y.; Sun W.; Ni J.; Kang X. TiO2 Gas Sensors Combining Experimental and DFT Calculations: A Review. Nanomaterials 2022, 12 (20), 3611 10.3390/nano12203611. 36296801 PMC9607066

[ref16] Gutiérrez J.; Robein Y. N.; Juan J.; Di Nezio M. S.; Pistonesi C.; González E. A.; Santos R.; Pistonesi M. F. A Combined Experimental and DFT Study on the Zero Valent Iron/Reduced Graphene Oxide Doped QCM Sensor for Determination of Trace Concentrations of As Using a Flow-Batch System. Sens. Actuators, B 2024, 404, 135233 10.1016/j.snb.2023.135233.

[ref17] Arunragsa S.; Seekaew Y.; Pon-On W.; Wongchoosuk C. Hydroxyl Edge-Functionalized Graphene Quantum Dots for Gas-Sensing Applications. Diamond Relat. Mater. 2020, 105, 107790 10.1016/j.diamond.2020.107790.

[ref18] Facure M. H. M.; Schneider R.; Mercante L. A.; Correa D. S. Rational Hydrothermal Synthesis of Graphene Quantum Dots with Optimized Luminescent Properties for Sensing Applications. Mater. Today Chem. 2022, 23, 100755 10.1016/j.mtchem.2021.100755.

[ref19] Zhao Y.; Truhlar D. G. The M06 Suite of Density Functionals for Main Group Thermochemistry, Thermochemical Kinetics, Noncovalent Interactions, Excited States, and Transition Elements: Two New Functionals and Systematic Testing of Four M06-Class Functionals and 12 Other Functionals. Theor. Chem. Acc. 2008, 120 (1–3), 215–241. 10.1007/s00214-007-0310-x.

[ref20] Frisch M. J.; Trucks G. W.; Schlegel H. B.; Scuseria G. E.; Robb M. A.; Cheeseman J. R.; Scalmani G.; Barone V.; Mennucci B.; Petersson G. A.; Nakatsuji H.; Caricato M.; Li X.; Hratchian H. P.; Izmaylov A. F.; Bloino J.; Zheng G.; Sonnenberg J. L.; Hada M.; Ehara M.; Toyota K.; Fukuda R.; Hasegawa J.; Ishida M.; Nakajima T.; Honda Y.; Kitao O.; Nakai H.; Vreven T.; Montgomery J. A. Jr.; Peralta J. E.; Ogliaro F.; Bearpark M.; Heyd J. J.; Brothers E.; Kudin K. N.; Staroverov V. N.; Keith T.; Kobayashi R.; Normand J.; Raghavachari K.; Rendell A.; Burant J. C.; Iyengar S. S.; Tomasi J.; Cossi M.; Rega N.; Millam J. M.; Klene M.; Knox J. E.; Cross J. B.; Bakken V.; Adamo C.; Jaramillo J.; Gomperts R.; Stratmann R. E.; Yazyev O.; Austin A. J.; Cammi R.; Pomelli C.; Ochterski J. W.; Martin R. L.; Morokuma K.; Zakrzewski V. G.; Voth G. A.; Salvador P.; Dannenberg J. J.; Dapprich S.; Daniels A. D.; Farkas O.; Foresman J. B.; Ortiz J. V.; Cioslowski J.; Fox D. J. Gaussian 09, Revision D.01; Gaussian, Inc: Wallingford, CT, 2013.

[ref21] Jmol: An Open-Source Java Viewer for Chemical Structures in 3D. Http://Www.Jmol.Org/.

[ref22] Lu T.; Chen F. Multiwfn: A Multifunctional Wavefunction Analyzer. J. Comput. Chem. 2012, 33 (5), 580–592. 10.1002/jcc.22885. 22162017

[ref23] Andre R. S.; Pereira J. C.; Mercante L. A.; Locilento D.; Mattoso L. H. C.; Correa D. S. ZnO-Co3O4 Heterostructure Electrospun Nanofibers Modified with Poly(Sodium 4-Styrenesulfonate): Evaluation of Humidity Sensing Properties. J. Alloys Compd. 2018, 767, 1022–1029. 10.1016/j.jallcom.2018.07.132.

[ref24] Wang P.; Wang T.; Li F.; Li D.; Yang Y.; Yu H.; Dong X. Enhanced Sensing Response of the First Polyoxometalate Electron Acceptor Modified MoS2 for NO2 Gas Detection at Room Temperature. Sens. Actuators, B 2023, 382, 133495 10.1016/j.snb.2023.133495.

[ref25] de Menezes R. F.; Pirani F.; Coletti C.; de Macedo L. G. M.; Gargano R. Functionalized Graphene-Based Quantum Dots: Promising Adsorbents for CO, NO2, SO2, and NH3 Pollutant Gases. Mater. Today Commun. 2022, 31, 103426 10.1016/j.mtcomm.2022.103426.

[ref26] Grabowski S. J. Ab Initio Calculations on Conventional and Unconventional Hydrogen BondsStudy of the Hydrogen Bond Strength. J. Phys. Chem. A 2001, 105 (47), 10739–10746. 10.1021/jp011819h.

[ref27] Koch U.; Popelier P. L. A. Characterization of C-H-O Hydrogen Bonds on the Basis of the Charge Density. 1995; Vol. 99 https://pubs.acs.org/sharingguidelines.

[ref28] Prasert K.; Sutthibutpong T. Unveiling the Fundamental Mechanisms of Graphene Oxide Selectivity on the Ascorbic Acid, Dopamine, and Uric Acid by Density Functional Theory Calculations and Charge Population Analysis. Sensors 2021, 21 (8), 2773 10.3390/s21082773. 33920002 PMC8071017

[ref29] Arunan E.; Desiraju G. R.; Klein R. A.; Sadlej J.; Scheiner S.; Alkorta I.; Clary D. C.; Crabtree R. H.; Dannenberg J. J.; Hobza P.; Kjaergaard H. G.; Legon A. C.; Mennucci B.; Nesbitt D. J. Definition of the Hydrogen Bond (IUPAC Recommendations 2011). Pure Appl. Chem. 2011, 83 (8), 1637–1641. 10.1351/PAC-REC-10-01-02.

[ref30] Choi Y. R.; Yoon Y. G.; Choi K. S.; Kang J. H.; Shim Y. S.; Kim Y. H.; Chang H. J.; Lee J. H.; Park C. R.; Kim S. Y.; Jang H. W. Role of Oxygen Functional Groups in Graphene Oxide for Reversible Room-Temperature NO2 Sensing. Carbon 2015, 91, 178–187. 10.1016/j.carbon.2015.04.082.

[ref31] Fatima Q.; Haidry A. A.; Yao Z.; He Y.; Li Z.; Sun L.; Xie L. The Critical Role of Hydroxyl Groups in Water Vapor Sensing of Graphene Oxide. Nanoscale Adv. 2019, 1 (4), 1319–1330. 10.1039/C8NA00135A. 36132612 PMC9473244

[ref32] Zhang C.; Wu K.; Liao H.; Debliquy M. Room Temperature WO3-Bi2WO6 Sensors Based on Hierarchical Microflowers for Ppb-Level H2S Detection. Chem. Eng. J. 2022, 430, 132813 10.1016/j.cej.2021.132813.

[ref33] Wu K.; He X.; Ly A.; Lahem D.; Debliquy M.; Zhang C. Highly Sensitive and Selective Gas Sensors Based on 2D/3D Bi2MoO6Micro-Nano Composites for Trimethylamine Biomarker Detection. Appl. Surf. Sci. 2023, 629, 157443 10.1016/j.apsusc.2023.157443.

[ref34] Chu X.; Wang J.; Gao Q.; Wang Y.; Liang S.; Bai L.; Dong Y.; Epifani M. High Selectivity Trimethylamine Sensors Based on Graphene-NiGa2O4 Nanocomposites Prepared by Hydrothermal Method. Phys. E (Amsterdam, Neth.) 2020, 118, 113788 10.1016/j.physe.2019.113788.

[ref35] Zhou L.; Mi Q.; Jin Y.; Li T.; Zhang D. Construction of MoO3/MoSe2 Nanocomposite-Based Gas Sensor for Low Detection Limit Trimethylamine Sensing at Room Temperature. J. Mater. Sci.:Mater. Electron. 2021, 32 (13), 17301–17310. 10.1007/s10854-021-06242-5.

[ref36] Meng D.; Qiao T.; Wang G.; Shen Y.; San X.; Pan Y.; Meng F. NiO-Functionalized In2O3 Flower-like Structures with Enhanced Trimethylamine Gas Sensing Performance. Appl. Surf. Sci. 2022, 577, 151877 10.1016/j.apsusc.2021.151877.

[ref37] Meng D.; Li R.; zhang L.; Wang G.; Zhang Y.; San X.; Wang X. Synthesis of NiMoO4-Functionalized MoO3 Nanorods with Enhanced TMA Gas Sensing Properties. Sens. Actuators Rep. 2022, 4, 100104 10.1016/j.snr.2022.100104.

[ref38] Ji Y.; Zhang N.; Xu J.; Jin Q.; San X.; Wang X. Co3O4/In2O3 p-n Heterostructures Based Gas Sensor for Efficient Structure-Driven Trimethylamine Detection. Ceram. Int. 2023, 49 (11), 17354–17362. 10.1016/j.ceramint.2023.02.103.

[ref39] Yang Y.; Xie Y.; Wang Q.; Wu X. Inhibition of Lysozyme Fibrillation by Functional Groups in Graphene Oxide Quantum Dots. Chem. Phys. Lett. 2022, 801, 139749 10.1016/j.cplett.2022.139749.

[ref40] Kappen J.; Aravind M. K.; Varalakshmi P.; Ashokkumar B.; John S. A. Hydroxyl Rich Graphene Quantum Dots for the Determination of Hg(II) in the Presence of Large Concentration of Major Interferents and in Living Cells. Microchem. J. 2020, 157, 104915 10.1016/j.microc.2020.104915.

