# Supplementary material for: Theoretical and Experimental Insights into the Chemiresistive Sensing Response of Graphene Quantum Dots: The Role of Oxygen Functional Groups
Source: ACS Omega. 2025 Feb 21;10(8):7831–8. doi: 10.1021/acsomega.4c08588 (PMC11886424; doi:10.1021/acsomega.4c08588)
Supplement: Supplementary file 1 — ao4c08588_si_001.pdf [file ao4c08588_si_001.pdf]

Supplementary Material

Theoretical and experimental insights into the chemiresistive sensing response of graphene quantum dots: the role of oxygen functional groups

Bruno S. Sampaio[1], Murilo H. M. Facure[2], Rafaela S. Andre[2], Daniel S. Correa[2], Tiago V. Alves[1]*, Luiza A. Mercante[1]*

[1]Instituto de Química, Universidade Federal da Bahia (UFBA), 40170-115, Salvador, BA, Brazil

[2]Nanotechnology National Laboratory for Agriculture (LNNA), Embrapa Instrumentation, 13560-970, São Carlos, SP, Brazil

*Corresponding authors:

tiagova@ufba.br (T.V. Alves)

lmercante@ufba.br (L.A. Mercante)

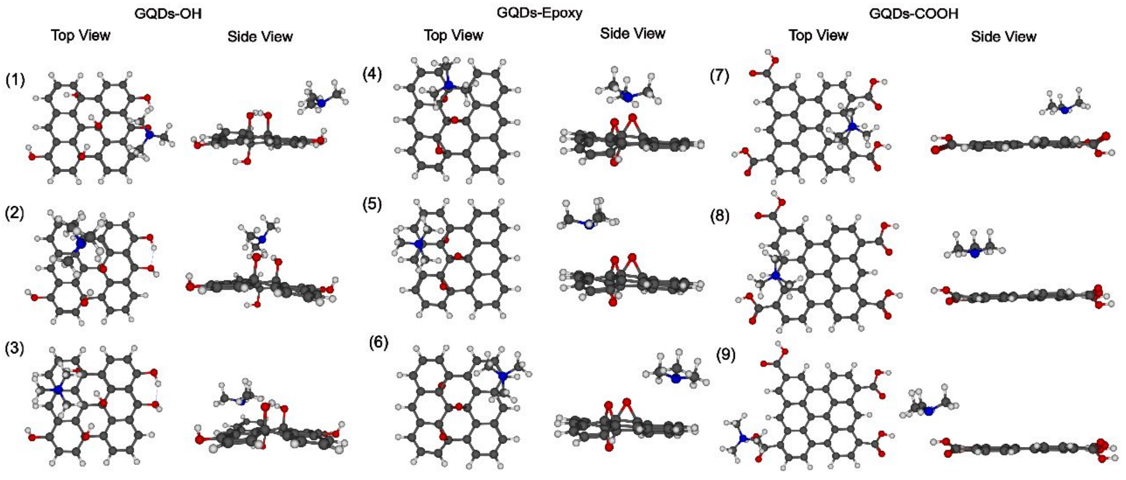

Figure S1. Top and side view of all scans between TMA and the three GQDs models.

Table S1. Cartesian Coordinates, lowest frequency values, and electronic energy values for the computationally modeled structures of the three GQDs and TMA.

GQDs-OH Lowest Freq. = 25.43 cm^{-1}				GQDs-Epoxy Lowest Freq. = 45.67 cm^{-1}			
E = -1527.84989925 a.u				E = - 1300.51789533 a.u.			
6	-4.199629000	-2.116161000	-0.516388000	6	-3.606477000	3.045255000	-0.308286000
6	-4.377533000	-0.747659000	-0.395818000	6	-2.409414000	3.710446000	-0.146470000
6	-3.252461000	0.102135000	-0.226474000	6	-1.182070000	3.001965000	0.004810000
6	-2.967187000	-2.734169000	-0.365477000	6	-3.654487000	1.642753000	-0.332164000
6	-1.833002000	-1.975750000	0.239845000	6	-2.473705000	0.910802000	-0.171288000
6	-1.950571000	-0.492369000	-0.054793000	6	-1.251058000	1.574295000	0.001633000
6	-0.442378000	-2.529420000	-0.099226000	6	-2.507769000	-0.593149000	-0.275394000
6	-0.270705000	-3.911258000	-0.325201000	6	-3.778108000	-1.318938000	-0.051419000
6	1.009302000	-4.445528000	-0.443464000	6	-3.753429000	-2.657926000	0.201049000
6	0.675699000	-1.700610000	-0.019222000	6	-1.256488000	-1.372808000	-0.067726000
6	2.002403000	-2.242175000	-0.129692000	6	-1.309817000	-2.848448000	0.190369000
6	2.139647000	-3.644534000	-0.345054000	6	-2.564579000	-3.437555000	0.297384000
6	-2.246602000	2.348513000	-0.035703000	6	1.271427000	2.954793000	0.077777000
6	-3.362923000	1.507187000	-0.182160000	6	0.057178000	3.668422000	0.118406000
6	-0.830507000	0.343977000	0.006039000	6	-0.007928000	0.819858000	0.343638000
6	-0.951915000	1.742413000	-0.051076000	6	1.287805000	1.519216000	0.105836000
6	0.522173000	-0.251101000	0.391617000	6	-0.026911000	-0.653739000	0.417523000
6	1.713531000	0.618925000	0.009944000	6	1.265534000	-1.431109000	0.301755000
6	3.114231000	-1.399085000	-0.011800000	6	-0.081748000	-3.524235000	0.136939000
6	3.015784000	0.013987000	-0.024453000	6	1.167096000	-2.885608000	-0.030468000
6	-2.346836000	3.764003000	0.217785000	6	2.527400000	3.620370000	-0.032489000
6	-1.213473000	4.407495000	0.758495000	6	3.706113000	2.916132000	-0.159522000
6	0.048032000	3.846454000	0.709633000	6	3.706505000	1.514522000	-0.186273000
6	0.217804000	2.677311000	-0.224191000	6	2.497949000	0.818948000	-0.050689000
6	1.570952000	2.002252000	-0.152180000	6	2.544614000	-0.681435000	0.021327000
6	2.697954000	2.813057000	-0.315660000	6	3.616560000	-1.431172000	-0.664060000
6	4.138243000	0.884220000	-0.151844000	6	2.335953000	-3.522931000	-0.425919000
6	3.976028000	2.248102000	-0.291890000	6	3.501559000	-2.782817000	-0.787502000
8	-5.670540000	-0.183139000	-0.555152000	1	-2.380773000	4.799298000	-0.137210000
1	-5.788270000	0.420827000	0.189686000	1	-4.528130000	3.614146000	-0.427727000
8	4.406111000	-2.021843000	-0.019000000	1	-4.602747000	1.142161000	-0.483498000
1	4.460623000	-2.546140000	0.791813000	1	0.079831000	4.755654000	0.076594000
8	5.435933000	0.386731000	-0.174159000	1	-4.717932000	-0.782920000	-0.070020000
1	5.346439000	-0.583265000	-0.130581000	1	-4.706029000	-3.171305000	0.342203000
8	-1.920275000	-2.149226000	1.729539000	1	-2.636463000	-4.512966000	0.444908000
1	-1.209242000	-1.587868000	2.084179000	1	-0.101238000	-4.613493000	0.091417000
8	0.512596000	-0.303465000	1.879600000	1	2.310626000	-4.594653000	-0.611674000
1	0.305947000	0.605931000	2.146393000	1	4.642950000	0.974154000	-0.263325000
8	0.176692000	3.189137000	-1.623279000	1	2.533806000	4.709536000	-0.027301000
1	-0.687997000	3.624984000	-1.668610000	1	4.650393000	3.454125000	-0.240145000
1	-2.902788000	-3.811029000	-0.463174000	1	4.468363000	-0.903395000	-1.074892000
1	-5.073763000	-2.717950000	-0.781025000	1	4.322574000	-3.332950000	-1.249460000

1	-1.132326000	-4.565332000	-0.367692000	8	-1.685829000	-1.047778000	-1.393868000
1	1.128739000	-5.517454000	-0.615999000	8	-0.151600000	0.090920000	1.616729000
1	3.131465000	-4.069317000	-0.477631000	8	2.204246000	-1.167247000	1.334326000
1	4.870634000	2.858101000	-0.412434000				
1	2.560526000	3.876896000	-0.482155000				
1	0.920328000	4.329544000	1.140104000				
1	-1.346453000	5.376707000	1.251153000				
1	-3.336140000	4.208039000	0.317625000				
1	-4.348224000	1.970509000	-0.251593000				

GQDs-COOH

Lowest Freq. = 7.94 cm^{-1}

E = -1829.18348393 a.u.

6	-3.052902000	-3.613418000	-0.212857000
6	-3.660318000	-2.389992000	-0.080747000
6	-2.858294000	-1.195791000	-0.009630000
6	-1.652102000	-3.737588000	-0.198061000
6	-0.834760000	-2.635525000	-0.046696000
6	-1.428202000	-1.330547000	-0.001104000
6	0.625418000	-2.771037000	0.048636000
6	1.225935000	-4.005258000	0.197922000
6	2.624189000	-4.142787000	0.209584000
6	1.447604000	-1.596247000	0.004047000
6	2.885363000	-1.730735000	0.013172000
6	3.452069000	-3.054669000	0.082948000
6	-2.637070000	1.237734000	0.006121000
6	-3.422750000	0.082580000	-0.005610000
6	-0.613083000	-0.176707000	0.005789000
6	-1.203183000	1.110693000	0.011563000
6	0.842201000	-0.315745000	-0.005136000
6	1.651333000	0.838553000	-0.013855000
6	3.660696000	-0.569463000	0.003028000
6	3.073989000	0.697451000	-0.011179000
6	-3.218723000	2.559253000	0.008231000
6	-2.394069000	3.659594000	0.009525000
6	-0.995664000	3.528409000	0.003145000
6	-0.384244000	2.291225000	0.001753000
6	1.080220000	2.154061000	-0.006059000
6	1.918110000	3.249663000	-0.007502000
6	3.896077000	1.856909000	-0.013111000
6	3.327326000	3.102319000	-0.012837000
1	-3.676487000	-4.494872000	-0.317628000
1	-1.229558000	-4.728184000	-0.311129000
1	-4.493164000	0.189700000	-0.017440000
1	0.627481000	-4.900640000	0.309505000
1	3.072725000	-5.125528000	0.307049000
1	4.739481000	-0.633100000	0.013637000
1	4.977698000	1.758114000	-0.013962000
1	-0.406879000	4.436797000	-0.002132000
1	-2.828357000	4.651725000	0.012290000
1	1.524537000	4.257381000	-0.002913000
6	-4.690878000	2.790985000	0.006086000
8	-5.565388000	1.953657000	-0.035322000
8	-5.006138000	4.105785000	0.055208000
1	-5.973642000	4.153246000	0.044213000
6	4.906446000	-3.394799000	0.042727000
8	5.356058000	-4.449070000	0.429436000
8	5.702680000	-2.457366000	-0.509119000
1	6.602233000	-2.820736000	-0.499262000
6	-5.155173000	-2.438852000	-0.042549000
8	-5.803301000	-3.340278000	-0.523302000
8	-5.732073000	-1.424681000	0.622593000
1	-6.691366000	-1.560548000	0.578562000
6	4.232091000	4.285036000	-0.014560000
8	5.438700000	4.228244000	-0.017320000
8	3.564594000	5.456458000	-0.012625000
1	4.228331000	6.162827000	-0.013884000

TMA

Lowest Freq. = 255.13 cm^{-1}

E = -174.389774581 a.u.

7	0.000005000	0.000013000	-0.387380000
6	1.137068000	0.781663000	0.062654000
1	1.058266000	1.803245000	-0.319460000
1	2.062631000	0.342395000	-0.319746000
1	1.204850000	0.828007000	1.167195000
6	-1.245482000	0.593894000	0.062651000
1	-1.327788000	1.615162000	-0.319553000
1	-1.319627000	0.629155000	1.167201000
1	-2.090790000	0.014949000	-0.319649000
6	0.108408000	-1.375549000	0.062645000
1	1.032565000	-1.818065000	-0.319418000
1	-0.734743000	-1.957532000	-0.319798000
1	0.114635000	-1.457450000	1.167187000

Table S2. Electronic energy with respect to distance in the scans for the nine selected adsorption sites of each structure.

GQDs-OH

(1)		(2)		(3)	
r (Å)	E (kcal/mol)	r (Å)	E (kcal/mol)	r (Å)	E (kcal/mol)
7.1	4.796E-14	7.1	-3.908E-14	7.1	-0.1369
6.9	-0.005472	6.9	0.01745	6.9	-0.04023
6.7	-0.05854	6.7	0.01736	6.7	-4.450E-06
6.5	-0.1443	6.5	-9.663E-04	6.5	-0.005150
6.3	-0.2491	6.3	-0.03567	6.3	-0.04335
6.1	-0.3753	6.1	-0.09558	6.1	-0.1327
5.9	-0.5448	5.9	-0.1846	5.9	-0.2417
5.7	-0.7694	5.7	-0.3001	5.7	-0.3265
5.5	-1.043	5.5	-0.4448	5.5	-0.4919
5.3	-1.350	5.3	-0.6454	5.3	-0.7304
5.1	-1.681	5.1	-0.8869	5.1	-0.9867
4.9	-2.035	4.9	-1.149	4.9	-1.244
4.7	-2.431	4.7	-1.408	4.7	-1.595
4.5	-2.888	4.5	-1.688	4.5	-1.986
4.3	-3.430	4.3	-2.048	4.3	-2.470
4.1	-4.031	4.1	-2.690	4.1	-3.176
3.9	-4.689	3.9	-3.268	3.9	-4.072
3.7	-5.426	3.7	-3.663	3.8	-4.565
3.5	-6.256	3.5	-4.638	3.75	-4.825
3.3	-7.256	3.3	-5.390	3.7	-5.092
3.1	-8.447	3.1	-6.298	3.65	-5.356
2.9	-9.911	2.9	-7.387	3.6	-5.617
2.8	-10.73	2.8	-7.979	3.55	-5.882
2.7	-11.56	2.75	-8.262	3.5	-6.150
2.65	-11.98	2.7	-8.531	3.45	-6.406
2.6	-12.39	2.65	-8.789	3.4	-6.628
2.55	-12.79	2.6	-9.036	3.35	-6.802
2.5	-13.16	2.55	-9.270	3.3	-6.921
2.45	-13.48	2.5	-9.488	3.25	-6.987
2.4	-13.76	2.45	-9.686	3.2	-7.004
2.35	-13.97	2.4	-9.860	3.15	-6.967
2.3	-14.10	2.35	-10.01	3.1	-6.854
2.25	-14.13	2.3	-10.13	3.05	-6.628
2.2	-14.04	2.25	-10.20	3	-6.252
2.15	-13.80	2.2	-10.23	2.95	-5.700
2.1	-13.38	2.15	-10.19	2.9	-4.951
2.05	-12.77	2.1	-10.07	2.85	-3.976
2	-11.91	2.05	-9.847	2.8	-2.737
1.95	-10.78	2	-9.500	2.7	0.7423
1.9	-9.332	1.95	-9.011		
1.85	-7.512	1.9	-8.358		
1.8	-5.263	1.85	-7.511		
1.75	-2.523	1.8	-6.444		
1.7	0.7839				

GQDs-Epoxy

(4)		(5)		(6)	
r (Å)	E (kcal/mol)	r (Å)	E (kcal/mol)	r (Å)	E (kcal/mol)
7.1	0.000	7.1	-0.02290	7.1	-0.005076
6.9	-0.001186	6.9	-0.07169	6.9	-0.02632
6.7	-0.004989	6.7	-0.1327	6.7	-0.06108
6.5	-0.01338	6.5	-0.2000	6.5	-0.1044
6.3	-0.04276	6.3	-0.2678	6.3	-0.1620
6.1	-0.08590	6.1	-0.3453	6.1	-0.2406
5.9	-0.1128	5.9	-0.4184	5.9	-0.3369
5.7	-0.1633	5.7	-0.4970	5.7	-0.4422
5.5	-0.3012	5.5	-0.6024	5.5	-0.5650
5.3	-0.5173	5.3	-0.8090	5.3	-0.7451

5.1	-0.7461	5.1	-1.042	5.1	-0.9777
4.9	-1.059	4.9	-1.342	4.9	-1.224
4.7	-1.515	4.7	-1.739	4.7	-1.519
4.5	-1.995	4.5	-2.213	4.5	-1.865
4.3	-2.644	4.3	-2.715	4.3	-2.225
4.1	-3.580	4.1	-3.339	4.1	-2.667
3.9	-4.833	3.9	-4.122	3.9	-3.167
3.8	-5.568	3.7	-5.074	3.7	-3.828
3.75	-5.938	3.6	-5.604	3.6	-4.178
3.7	-6.311	3.55	-5.891	3.55	-4.380
3.65	-6.700	3.5	-6.188	3.5	-4.589
3.6	-7.112	3.45	-6.485	3.45	-4.793
3.55	-7.537	3.4	-6.765	3.4	-4.985
3.5	-7.951	3.35	-7.027	3.35	-5.161
3.45	-8.337	3.3	-7.278	3.3	-5.323
3.4	-8.677	3.25	-7.523	3.25	-5.475
3.35	-8.959	3.2	-7.755	3.2	-5.611
3.3	-9.182	3.15	-7.959	3.15	-5.720
3.25	-9.338	3.1	-8.113	3.1	-5.781
3.2	-9.388	3.05	-8.191	3.05	-5.774
3.15	-9.343	3	-8.174	3	-5.693
3.1	-9.146	2.95	-8.015	2.95	-5.532
3.05	-8.759	2.9	-7.778	2.9	-5.279
3	-8.142	2.85	-7.414	2.85	-4.912
2.95	-7.256	2.8	-6.897	2.8	-4.401
2.9	-6.060	2.75	-6.180	2.75	-3.707
2.85	-4.515	2.7	-5.224	2.7	-2.796
2.8	-2.571	2.65	-3.995	2.65	-1.649
2.7	2.789	2.6	-2.467	2.6	-0.2326
2.5	22.00	2.5	1.679	2.5	3.592
				2.3	16.96

GQDs-COOH

(7)		(8)		(9)	
r (Å)	E (kcal/mol)	r (Å)	E (kcal/mol)	r (Å)	E (kcal/mol)
7.1	-0.009519	7.1	-0.003828	7.1	0.002999
6.9	-3.553E-15	6.9	-0.01094	6.9	0.004863
6.7	-0.001493	6.7	-0.02857	6.7	0.004286
6.5	-0.02188	6.5	-0.06094	6.5	-4.204E-04
6.3	-0.07179	6.3	-0.1167	6.3	-0.01019
6.1	-0.1574	6.1	-0.1989	6.1	-0.02528
5.9	-0.2717	5.9	-0.3021	5.9	-0.04772
5.7	-0.4086	5.7	-0.4289	5.7	-0.07756
5.5	-0.5942	5.5	-0.5979	5.5	-0.1120
5.3	-0.8300	5.3	-0.7925	5.3	-0.1559
5.1	-1.080	5.1	-1.026	5.1	-0.2116
4.9	-1.370	4.9	-1.289	4.9	-0.2678
4.7	-1.725	4.7	-1.580	4.7	-0.3409
4.5	-2.094	4.5	-1.937	4.5	-0.4333
4.3	-2.506	4.3	-2.285	4.3	-0.5317
4.1	-2.996	4.1	-2.734	4.1	-0.6627
3.9	-3.658	3.9	-3.291	3.9	-0.8100
3.7	-4.415	3.8	-3.588	3.7	-0.9855
3.6	-4.884	3.75	-3.745	3.5	-1.223
3.55	-5.159	3.7	-3.909	3.3	-1.529
3.5	-5.442	3.65	-4.083	3.1	-1.973
3.45	-5.713	3.6	-4.273	2.9	-2.485
3.4	-5.960	3.55	-4.481	3	-2.227
3.35	-6.187	3.5	-4.694	2.95	-2.355
3.3	-6.408	3.45	-4.884	2.9	-2.485
3.25	-6.625	3.4	-5.031	2.85	-2.613

3.2	-6.826	3.35	-5.134	2.8	-2.734
3.15	-6.986	3.3	-5.194	2.75	-2.843
3.1	-7.082	3.25	-5.212	2.7	-2.933
3.05	-7.095	3.2	-5.182	2.65	-3.002
3	-7.021	3.15	-5.079	2.6	-3.041
2.95	-6.854	3.1	-4.872	2.55	-3.045
2.9	-6.570	3.05	-4.537	2.5	-3.001
2.85	-6.140	3	-4.054	2.45	-2.896
2.8	-5.524	2.95	-3.405	2.4	-2.708
2.75	-4.689	2.9	-2.570	2.35	-2.421
2.7	-3.603	2.85	-1.514	2.3	-2.013
2.65	-2.230	2.8	-0.1900	2.25	-1.462
2.6	-0.5284	2.7	3.471	2.2	-0.7390
2.5	4.084	2.5	16.29	2.15	0.1887
				2.1	1.367
				2.05	2.837
				2	4.655
				1.9	9.602
				1.7	27.08

Table S3. Cartesian coordinates of the GQDs-OH (1) and (2) after optimization at the equilibrium distance of the rigid scan.

GQDs-OH (1) – Optimized			
E = -1702.27062640 a.u			
6	-3.985724363	-3.144761887	-0.435395823
6	-4.535241363	-1.933394887	-0.047342823
6	-2.622014363	-3.335631887	-0.599668823
1	-4.674562363	-3.964541887	-0.657928823
6	-3.709559363	-0.783510887	0.062505177
8	-5.940573363	-1.810407887	0.110739177
6	-2.284547363	-0.933381887	-0.093650823
6	-4.224174363	0.497832113	0.348299177
6	-1.669409363	-2.318376887	-0.067894823
1	-2.256621363	-4.302425887	-0.923033823
6	-0.276378363	-2.359011887	-0.707895823
8	-1.396304363	-2.661501887	1.372652177
6	-1.483560363	0.213838113	-0.099418823
6	0.267452637	-3.590759887	-1.121772823
6	0.525050637	-1.215689887	-0.713309823
6	1.606025637	-3.669091887	-1.507826823
1	-0.335989363	-4.489150887	-1.086800823
6	2.428490637	-2.552975887	-1.497688823
1	2.018217637	-4.632975887	-1.814505823
6	1.907536637	-1.287877887	-1.098080823
6	0.031956637	0.060344113	-0.069337823
6	2.715580637	-0.144299887	-1.030521823
1	3.470671637	-2.619205887	-1.798070823
6	-3.409610363	1.640835113	0.423431177
6	-2.027778363	1.495932113	0.084446177
6	-3.875114363	2.916305113	0.905635177
1	-5.293531363	0.618084113	0.527559177
6	-1.261522363	2.774273113	-0.149448823
6	0.793986637	1.301325113	-0.503243823
8	0.378238637	-0.129469887	1.381416177
6	2.181460637	1.153075113	-0.833611823
6	0.214835637	2.573981113	-0.417887823
8	4.124144637	-0.321193887	-1.181524823
6	2.950595637	2.343996113	-0.991788823
6	-2.900665363	3.845753113	1.325842177
1	-4.906115363	3.002130113	1.245278177
6	-1.570944363	3.735813113	0.967314177
1	-3.209916363	4.671191113	1.975858177

1	-0.815304363	4.439888113	1.303009177
8	-1.755696363	3.363997113	-1.424742823
6	0.995043637	3.713465113	-0.628554823
6	2.364546637	3.590070113	-0.889941823
1	0.519860637	4.689117113	-0.598740823
8	4.301276637	2.279589113	-1.297645823
1	2.993438637	4.465200113	-1.049586823
1	-6.073275363	-1.363548887	0.956759177
1	4.390673637	-0.700822887	-0.316613823
1	4.511182637	1.322233113	-1.341553823
1	-0.851881363	-1.924812887	1.698836177
1	-0.051865363	0.619154113	1.825191177
1	-2.699928363	3.497288113	-1.250699823
7	4.171412637	-1.053761887	1.597742177
6	3.172680179	-2.067795239	1.881796149
6	5.452473637	-1.402481887	2.166538177
6	3.727270297	0.249659109	2.056302031
1	3.441232417	-3.002870242	1.382430515
1	3.090478805	-2.244115209	2.972167559
1	2.199916683	-1.744175348	1.501200630
1	6.194897101	-0.643118617	1.905719412
1	5.411735297	-1.478244540	3.270792753
1	5.784683738	-2.366652219	1.771714807
1	3.727153502	0.301209563	3.162691721
1	2.714497825	0.434969233	1.687756851
1	4.388098496	1.027150458	1.662999646
GQDs-OH (2) – Optimized			
E = -1702.25857330			
6	3.580331000	2.914543000	-0.930883000
6	3.918029000	1.582551000	-1.115347000
6	2.312476000	3.332282000	-0.561749000
1	4.355048000	3.661477000	-1.124560000
6	2.923726000	0.575841000	-1.004757000
8	5.232295000	1.236662000	-1.524299000
6	1.597484000	0.946146000	-0.577128000
6	3.199351000	-0.786442000	-1.246386000
6	1.315882000	2.345409000	-0.056223000
1	2.138654000	4.386891000	-0.380081000
6	-0.153665000	2.743296000	-0.263560000
8	1.451156000	2.322594000	1.446290000
6	0.604834000	-0.041352000	-0.519330000
6	-0.518463000	4.100871000	-0.369053000
6	-1.142095000	1.764510000	-0.173559000
6	-1.863294000	4.462816000	-0.330465000
1	0.244955000	4.863505000	-0.457387000
6	-2.864621000	3.511867000	-0.183744000
1	-2.137381000	5.516883000	-0.413652000
6	-2.529470000	2.128023000	-0.099625000
6	-0.759022000	0.317221000	0.078887000
6	-3.502237000	1.135652000	0.070925000
1	-3.910947000	3.807187000	-0.182768000
6	2.226165000	-1.792199000	-1.103489000
6	0.881307000	-1.385050000	-0.828321000
6	2.537757000	-3.197756000	-1.126644000
1	4.210668000	-1.088541000	-1.522020000
6	-0.186057000	-2.428900000	-1.042948000
6	-1.868250000	-0.664758000	-0.285116000
8	-0.598141000	0.244034000	1.545373000
6	-3.229437000	-0.244904000	-0.099420000
6	-1.581198000	-1.982348000	-0.659509000
8	-4.854118000	1.575751000	0.260989000
6	-4.241656000	-1.244435000	-0.191290000
6	1.568706000	-4.098026000	-0.632027000
1	3.573909000	-3.506204000	-1.254755000
6	0.255520000	-3.729814000	-0.427909000
1	1.883494000	-5.113981000	-0.370691000
1	-0.477257000	-4.407061000	-0.000005000

8	-0.304933000	-2.635879000	-2.515954000
6	-2.611679000	-2.918468000	-0.789207000
6	-3.932845000	-2.548049000	-0.524540000
1	-2.374707000	-3.922784000	-1.125987000
8	-5.581157000	-0.934439000	0.012922000
1	-4.752309000	-3.260727000	-0.610919000
1	5.513497000	0.521247000	-0.938746000
1	-4.874255000	2.031866000	1.113340000
1	-5.610397000	0.031554000	0.142204000
1	0.735933000	1.732648000	1.747566000
1	0.024328000	-0.485790000	1.736439000
1	0.586051000	-2.919339000	-2.769833000
7	1.571776633	-1.229511253	2.804043705
6	1.583552809	-2.680756653	2.817970670
6	2.796271078	-0.690273955	2.241679665
6	1.287696006	-0.661580420	4.109121344
1	1.736561436	-3.052595046	1.801015390
1	0.618402994	-3.055648294	3.169813952
1	2.379020362	-3.072394894	3.481752614
1	2.968605446	-1.133499960	1.256944586
1	3.658826510	-0.918477326	2.897956366
1	2.706145491	0.391441778	2.108856757
1	0.319647171	-1.020203246	4.469846619
1	1.245502790	0.428149550	4.028127954
1	2.062018218	-0.929855869	4.854244825

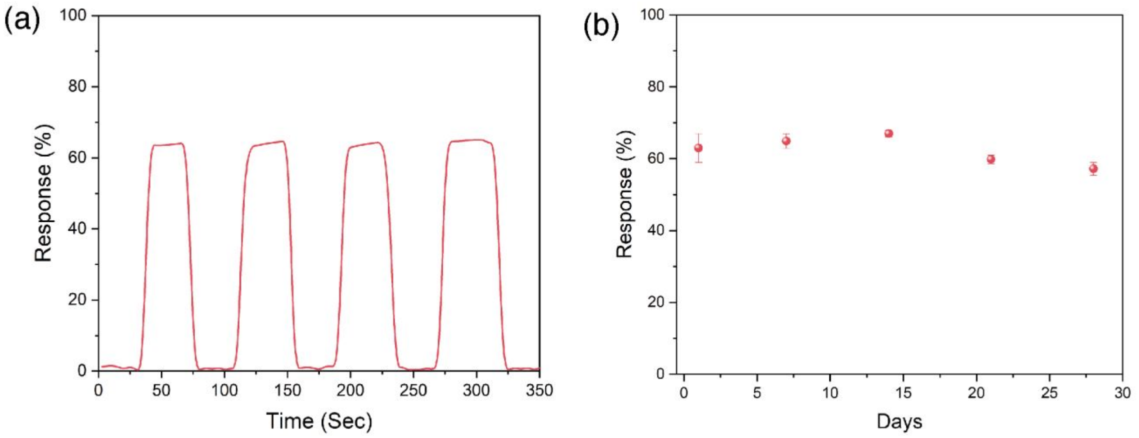

Figure S2. (a) Response stability of the GQDs-OH sensor towards 50 ppm TMA at room temperature and 50% RH. (b) Long-term stability of the GQDs-OH sensor.
